# Fast 3-D Imaging of Brain Organoids With a New Single-Objective Planar-Illumination Two-Photon Microscope

**DOI:** 10.3389/fnana.2019.00077

**Published:** 2019-08-20

**Authors:** Irina Rakotoson, Brigitte Delhomme, Philippe Djian, Andreas Deeg, Maia Brunstein, Christian Seebacher, Rainer Uhl, Clément Ricard, Martin Oheim

**Affiliations:** ^1^Centre National de la Recherche Scientifique (CNRS) UMR 8118, Brain Physiology Laboratory, Paris, France; ^2^Fédération de Recherche en Neurosciences CNRS FR 3636, Paris, France; ^3^Faculté de Sciences Fondamentales et Biomédicales, Université Paris Descartes, PRES Sorbonne Paris Cité, Paris, France; ^4^Master Program: MASTER Mention Biologie Cellulaire, Physiologie, Pathologies (BCPP), Spécialité Neurosciences, Université Paris Descartes - Paris 5, Paris, France; ^5^TILL id GmbH, Munich, Germany

**Keywords:** two photon, light sheet, brain organoids, hiPSC, stem cell, human disease modeling, spinning disc confocal

## Abstract

Human inducible pluripotent stem cells (hiPSCs) hold a large potential for disease modeling. hiPSC-derived human astrocyte and neuronal cultures permit investigations of neural signaling pathways with subcellular resolution. Combinatorial cultures, and three-dimensional (3-D) embryonic bodies (EBs) enlarge the scope of investigations to multi-cellular phenomena. The highest level of complexity, brain organoids that—in many aspects—recapitulate anatomical and functional features of the developing brain permit the study of developmental and morphological aspects of human disease. An ideal microscope for 3-D tissue imaging at these different scales would combine features from both confocal laser-scanning and light-sheet microscopes: a micrometric optical sectioning capacity and sub-micrometric spatial resolution, a large field of view and high frame rate, and a low degree of invasiveness, i.e., ideally, a better photon efficiency than that of a confocal microscope. In the present work, we describe such an instrument that uses planar two-photon (2P) excitation. Its particularity is that—unlike two- or three-lens light-sheet microscopes—it uses a single, low-magnification, high-numerical aperture objective for the generation and scanning of a virtual light sheet. The microscope builds on a modified Nipkow-Petráň spinning-disk scheme for achieving wide-field excitation. However, unlike the Yokogawa design that uses a tandem disk, our concept combines micro lenses, dichroic mirrors and detection pinholes on a single disk. This new design, advantageous for 2P excitation, circumvents problems arising with the tandem disk from the large wavelength difference between the infrared excitation light and visible fluorescence. 2P fluorescence excited by the light sheet is collected with the same objective and imaged onto a fast sCMOS camera. We demonstrate 3-D imaging of TO-PRO3-stained EBs and of brain organoids, uncleared and after rapid partial transparisation with triethanolamine formamide (RTF) and we compare the performance of our instrument to that of a confocal laser-scanning microscope (CLSM) having a similar numerical aperture. Our large-field 2P-spinning disk microscope permits one order of magnitude faster imaging, affords less photobleaching and permits better depth penetration than a confocal microscope with similar spatial resolution.

## Introduction

The development of pharmacological treatments for neuropsychiatric and neurodegenerative diseases has been hampered by the poor availability of preclinical models that adequately capture the complexity of human disorders (Gonzalez et al., 2017).

Human inducible pluripotent stem cells (hIPSCs) offer a promising platform for disease modeling and drug screening. A comparably new technique is the directed differentiation and reprogramming of patient fibroblasts into neurons, astrocytes, microglia and oligodendro-cytes. Their combinational culture permits the growth of embryonic bodies (EBs) and brain organoids, 3-D cultures that—in many aspects—recapitulate the development of the human brain (Fatehullah et al., 2016; Giandomenico and Lancaster, 2017). Together, hIPSCs, EBs, and brain organoids enable observations and experiments that were previously inconceivable, neither on human subjects nor in animal models (Lancaster et al., 2013; Kelava and Lancaster, 2016). Recent reports of functional, fully vascularized brain organoids have spurred hopes of growing even larger 3-D cell assemblies (Mansour et al., 2018), bringing the hitherto theoretical “*brain in a vat*”^1^ within reach of the imaginable.

Elucidation of neural circuit (dys-)function would benefit from the detailed, 3-D visualization of the fine structure of neurons, astrocytes and blood vessels over large fields of view and deep in tissue. Large-scale neuroanatomical imaging has become possible in cleared tissue sections (Wan et al., 2018), brain organoids (Renner et al., 2017) or even entire brains (Hama et al., 2015), but in many cases the resolution is rather at the level of cell bodies that at the synaptic scale. In addition to the difficulties associated with transparisation and tissue shrinking, imaging of large tissue volumes at spatial high-resolution presents considerable challenges: confocal and two-photon (2P) laser scanning microscopies set the “gold-standard” for diffraction-limited fluorescence imaging, but—being in most of their implementations point-scanning, i.e., sequential techniques—the image acquisition is often painstakingly slow. The reconstruction of large volumes at high spatial resolution often requires hours if not days of recording, putting high demands on the mechanical stability of the microscope, photostablity of the used fluorescent dyes, and incurring considerable cost for beam time. Line- and multi-spot scanning schemes overcome these limitations by parallelizing *excitation*, but they trade off resolution against speed and having relatively small fields of view, they require image stitching for larger views.

On the other end, selective-plane illumination microscopes (SPIM; Huisken et al., 2004) or light-sheet microscopes (Keller et al., 2008) decouple fluorescence excitation and collection by using orthogonal illumination and detection optical paths. Light-sheet microscopes have established themselves as efficient workhorses for volume imaging in cleared tissue. For example, a recent study used the 3DISCO/iDISCO cleaning method and conventional 1P light-sheet imaging to visualize and analyze early human development (Belle et al., 2017). In light-sheet microscopies, it is the parallelization of *both* *excitation* and fluorescence *detection* that allows for rapid 3-D imaging (Santi, 2011; Stelzer, 2015). However, one consequence of the lower-NA illumination and a result of excitation-light scattering in not perfectly transparent samples, is that the axial resolution of light-sheet microscopes remains poor compared to the optical sectioning achieved by spot-scanning microscopes. Improvement has been made with 2P light-sheet excitation (Truong et al., 2011; Mahou et al., 2014), by combining 2P-line excitation and confocal slit detection (Baumgart and Kubitscheck, 2012), by the use of Airy- (Vettenburg et al., 2014) or Bessel-beams for excitation (Fahrbach et al., 2013; Zhao et al., 2014; Müllenbroich et al., 2018), or a combination of these techniques (Andilla et al., 2017; Elisa et al., 2018). However, many of these recent techniques are not yet commercial and they afford considerable cost and complexity compared to standard 1- and 2P-laser scanning microscopes.

Another limitation of light-sheet microscopes results from the orthogonal arrangement of excitation and collection objectives: the need for non-standard procedures for embedding and holding the sample. Variants of light-sheet microscopes in which both illumination and detection objectives are mounted at an oblique angle with respect to the tissue surface and the sample half-space is left free exist (Holekamp et al., 2008; Chen et al., 2014; McGorty et al., 2015), but they have remained comparably marginal, and they are typically limited to short working distances and small fields of view.

An ideal microscope for volume imaging in cleared brain tissue (Reynaud et al., 2015) would combine speed, a sub-μm lateral and μm-axial resolution, a mm-field of view, an excitation depth of a few mm, a certain robustness to imperfect sample clearing and a large free space under the objective to accommodate a variety of different samples.

Here, we present a microscope with both excitation- and detection-parallelization that gets close to this ideal by combining advantages of 2P laser-scanning and light-sheet techniques. Our On-axis 2-photon virtual light-sheet generation *in vivo* imaging system (OASIS) uses a vast array of micro lenses arranged in four nested spirals on a single spinning disk (SD) to simultaneously scan ~40 independent excitation spots across the focal plane of a single, long-working distance, low-magnification, high-NA objective (Oheim et al., 2001). Rotation of the disk at 5,000 rpm results in rapid multi-spot scanning and creates a planar excitation in the focal plane. The fluorescence generated in each of the excitation spots is imaged through the same objective onto a pinhole in the center of each micro lens. With the remainder of the lens made opaque to out-of-focus and scattered fluorescence by a dichroic coating sparing only the tiny pinhole, only fluorescence emanating from the focal plane is detected. Each pinhole is imaged onto a large-format scientific Complementary Metal Oxide Semiconductor (sCMOS) camera, allowing near-diffraction limited imaging over a large field of view. This patented design, combining micro lenses and perforated dichroic mirrors on a single-SD, allowed us to retain the in-line, single-objective geometry of a classical microscope without the requirement for orthogonal illumination. As a consequence, our OASIS microscope is more versatile than two- or three-objective designs, and it allows the use of a low-mag, high-NA lens that simultaneously offers high spatial resolution and a large field-of-view. With its compact footprint (43 cm by 12 cm, or 17″ × 5″), it can accommodate large samples (cells, slices, explants and even entire animals, *in vivo*) without requiring tedious mounting procedures or relying on special sample holders. The OASIS concept combines the optical sectioning, spatial resolution and field-of-view of a 2P-scanning microscope with the speed of a light-sheet microscope. Due to 2P excitation, out-of-focus fluorescence excitation, out-of-focus photo-bleaching and photodamage are much reduced compared to a classical confocal microscope. We here describe this new microscope and compare it to a confocal laser-scanning microscope (CLSM) for imaging non-cleared and RTF-clarified brain organoids with nuclear staining.

## Materials and Methods

### Ethics Statement

All experimental procedures were performed in accordance with the French legislation and in compliance with the European Community Council Directive of November 24, 1986 (86/609/EEC) for the care and use of laboratory animals. Protocols were approved by the local ethics committee.

### Sample Preparation

#### hiPSC Culture and Formation of Embryonic Bodies

Episomal human induced pluripotent stem cells (hIPSCs, Gibco) were cultivated on mitomycin-treated mouse embryonic fibroblasts using DMEM/F12 medium (Invitrogen), supplemented with 10% knockout serum (Gibco). When hIPSCs had reached about 80% confluence, they were detached with versene (ThermoFisher). Cell aggregates were removed and a single-cell suspension obtained with a cell strainer having a 100-μm mesh size (Corning). For EB formation, 10^4^ cells were inoculated in 100 μl in each well of an ultra-low attachment, round-bottomed 96-well plate (Corning) and cultivated during 9 days in EB formation medium (StemCell Technologies).

#### Mouse Embryos

Embryos were of age E10.5 and E14.5. Mice were killed by cervical dislocation, the abdominal cavity was opened and the uterine horns were removed. Embryos were collected under a macroscope (Nikon SMZ800) and immersed in formalin (buffered 10% formaldehyde, VWR) overnight. They then were stored at 4°C in PBS/Sodium Azide 0, 02%. E10.5 embryos were used for whole-embryo cleaning; E14.5 embryos were embedded in Optimal Cutting Temperature (OCT) compound and sliced into 7-μm-thin sections on a cryotome (Cryocut 1800, Leica).

### Staining, Clearing and Embedding

#### Nuclear Staining

The samples were permeabilized by a 0.2% TritonX100 solution in PBS (during 15 min for 7-μm-thin embryo slices and EBs, 20 min for brain organoids, 1 h for the whole-mount E10.5 embryo). They were then incubated overnight in a 1:1,000 solution of TO-PRO3 (Invitrogen) or of chloroform-purified MG (Merck) in PBS and finally washed in PBS. At this point, non-cleared samples were mounted in a PBS-filled chamber under a glass coverslip for microscopy. We used a home-made recording chamber, that was modified from chambers designed for imaging and available as 3-D printer templates^2^.

#### Clearing

After nuclear staining, samples were processed for one of the three following clearing methods: TDE (Aoyagi et al., 2015), Clear^T2^ (Kuwajima et al., 2013) or RTF (Yu et al., 2018).

#### TDE

Samples were successively immersed in increasing concentrations (20%, 40% and 60%) of TDE (Sigma) solutions in PBS. The duration of each incubation varied as a function of the sample size: 1 h for 7-μm embryo slices and for EBs, 3 h for a whole E10.5 embryo.

#### Clear^T2^

Samples were immersed successively in: (i) a 25% formamide (Sigma)/10% polyethylene glycol (PEG8000, Merck) solution in PBS (10 min for embryo slices and EBs, 30 min for a whole E10.5 embryo); (ii) a 50% formamide/20% PEG8000 solution in PBS (5 min for slices and EBs, 15 min for whole embryo); and (iii) a 50% formamide/20% PEG8000 solution in PBS (1 h for slices and for EBs, 3 h for a whole E10.5 embryo).

#### RTF

Samples were successively immersed in: (i) a 30% triethanolamine (TEA, Sigma)/40% formamide (Sigma) solution (15 min for slices and for EBs, 20 min for brain organoids, 3 h 20 min for a whole E10.5 embryo); (ii) a 60% TEA/25% formamide solution (25 min for slices and for EBs, 30 min for brain organoids, 5 h for embryo); and (iii) a 70% TEA/15% formamide solution (25 min for slices and for EBs, 30 min for brain organoids, 5 h for whole embryo). In either case, after clearing, samples were mounted under a glass coverslip in a chamber filled with the respective final solution.

### Microscopy

#### Confocal Microscopy

We used a Zeiss LSM 710 microscope with a ×63/1.4NA oil-immersion objective, for the acquisition of the confocal data shown in Figure 2 and Supplementary Figure S3, and a ×40/1.0NA water-immersion objective for tissue sections and thick samples (embryo, EB, brain organoid), respectively. For a fair comparison of the performance for imaging thick samples of the confocal and 2P-virtual light-sheet OASIS microscope, we set the confocal pinhole to 2 Airy units and the scanned area was restricted to 900 × 900 pixels with a zoom resulting in an effective pixel size of 0.182 μm, equivalent to the OASIS. We also acquired confocal images at 1 Airy for comparison. The laser powers delivered to the sample are indicated in each figure. For 1PEF, we excited TO-PRO3 using the 633-nm line of a HeNe gas laser (Lasos). Fluorescence was collected in between 646 nm and 725 nm.

**Figure 1:**
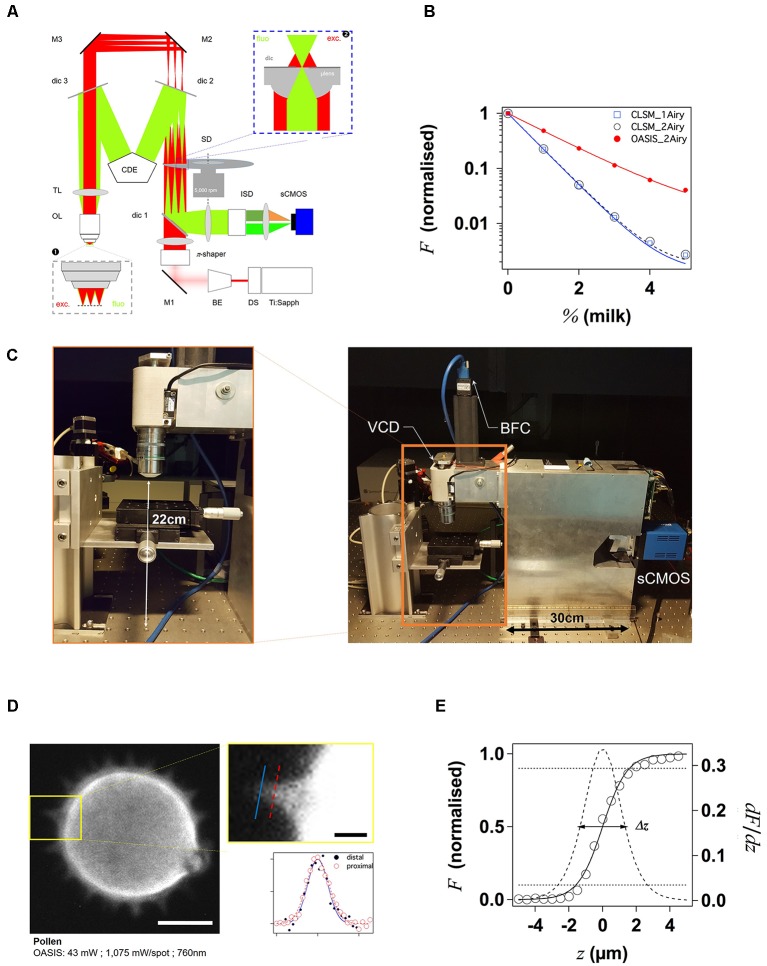
Principle and performance of the On-axis 2-photon virtual light-sheet generation in vivo imaging system (OASIS). **(A)** Simplified optical path of our custom 2P microscope. *Red*: infrared (IR) excitation, *green*: fluorescence. Ti:Sapph, fs-pulsed IR laser; DS, DeepSee module (pulse stretcher); BE, beam expander; π-shaper, optical element that converts the Gaussian beam into a top hat profile; M, mirrors; dic, dichroic mirrors; SD, spinning disk; TL, tube lens; CDE, corrective distance element; OL, objective lens; ISD, image-splitting device; sCMOS, camera. *Inset* ❶: (simplified) multi-spot excitation pattern and epi-collection of the generated fluorescence through the same objective. *Inset* ❷: detail of microlens/pinhole/dichroic coating arrangement. Note the offset between the excitation (exc.) and fluorescence foci (fluo.) at the level of the disk, produced by the CDE. **(B)** Measured depth penetration in turbid samples. Log-plot of 2P-excited fluorescence from a green-fluorescent Chroma test slide, topped either with water (0%) or increasing concentrations of milk (a model for the multi-scale scatterers present in tissue), for the OASIS (red dots) and a ZEISS LSM710. Confocal pinhole diameters were 1 and 2 Airy, as indicated. **(C)** OASIS prototype, note the compact size and space available around the objective, *inset*. VCD, voice coil *z*-drive; BFC, bright-field camera. **(D)** Assessment of OASIS lateral resolution. Equatorial section through an autofluorescent thorny pollen grain. Scale bar, 10 μm. *Inset*: magnified view of an in-focus spine and intensity profiles across the lines shown; Scale bar, 2 μm. **(E)** OASIS *z-* resolution. Axial-intensity profile measured from a *z*-stack of images acquired from a green fluorescent Chroma slide (solid line), and its derivative *dF*/*dz* (dashed). The 10%–90% intensity range was taken as axial optical sectioning capacity Δ*z*.

**Figure 2:**
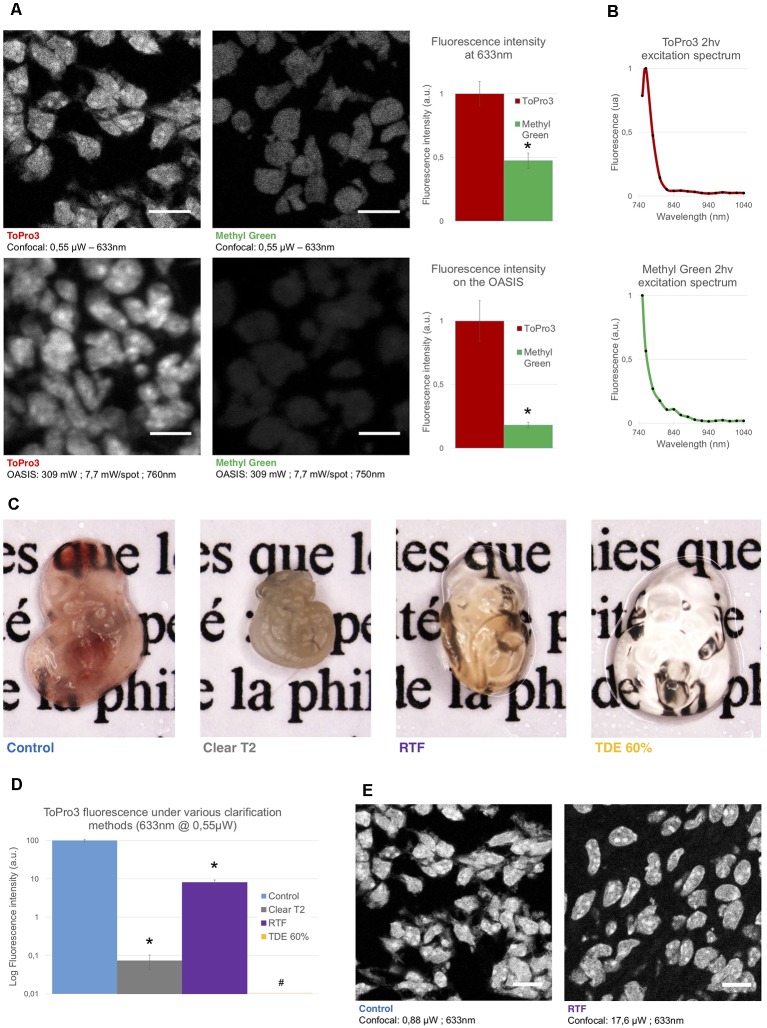
Comparison of the used nuclear stains and clearing methods. **(A)** *Left*, optical section of a slice from an E14.5 embryo, labeled with TO-PRO3 or Methyl Green (MG) and observed on a single-spot confocal laser scanning microscope confocal laser-scanning microscope (CLSM, *top*) or on the OASIS microscope (*bottom*). Scale-bar, 10 μm. *Right*, relative fluorescence intensity of TO-PRO3 and MG in nuclei of E14.5 embryo slices measured on a CLSM (*top right*) or on the OASIS (*bottom right*). **(B)** TO-PRO3 (*top*) and MG (*bottom*) 2P-excitation spectra. Color code as in **(A)**. **(C)** Macro-photographies of E10.5 embryos in control (PBS) and following three clearing protocols [Clear^T2^, rapid partial transparisation with triethanolamine formamide (RTF) and TDE 60%]. Note the variable degree of transparisation and the volume change. **(D)** Relative fluorescence of TO-PRO3-positive nuclei in E14.5 embryo slices observed on a CLSM after the same clearing protocols as above. Note the log-scale *P < 0.0001, ^#^Quantification impossible as the signal is too low. **(E)** Fluorescence loss upon clearing requires high laser powers. Confocal micrographs of slices from an E14.5 embryo labeled with TO-PRO3 under control (*left*) and after RTF clearing (*right*) along with the laser powers required to attain the same signal-to-background ratio. Scale-bar, 10 μm.

#### 2P-Spinning Disk Microscopy

We here give a brief overview of the OASIS microscope setup. A detailed description and full characterization of the system will be published elsewhere (Deeg et al., in preparation). 2PEF is excited in some 40 focused spots that are scanned simultaneously across the sample. The emission from each the spots is confocally filtered like in a Nipkov-Petráň Spinning Disk microscope (Kino, 1995). However, instead of two separate disks housing, respectively, aligned arrays of microlenses and confocal pinholes, we used a single newly engineered disk. For this purpose, a 2-mm thick glass disk was manufactured that features some 5,000 micro lenses (each having 666-μm diameter) on the front side, and a dielectric long-pass coating (715LP) on the rear side. The micro lenses are arranged in four nested spirals. The dielectric coating on the rear side of the disk has 5,000 pinholes (each consisting of a non-coated spot of 60 μm diameter co-axially aligned with one of the microlenses on the front side). For the used Nikon ×25/1.1NA objective, this corresponds to 2 Airy units.

For 2-photon excitation, we expanded an infrared (IR) femtosecond Gaussian laser beam of a Titanium Saphire laser (Spectra-Physics Mai Tai^®^ DeepSee) five times and collimated it to a beam diameter of about 5 mm that was aligned on the rear side of the disk as shown in Figure 1A. The disk transmits the IR light and the micro lenses generate a pattern of foci 7.8 mm in front of the disk. This pattern is imaged into the sample plan *via* the tube lens (TL; *f* = 200 mm) and objective (Nikon ×25/1.1NA, water). The fluorescence generated in each focal spot is then collected by the same objective. However, on the way back, between the TL and the disk two dichroic short-pass mirrors (custom design, SP705 Alluxa, Santa Rosa, CA, USA) are used to introduce a corrective distance, so that the emission of the excitation spots is imaged onto the pinholes of the disk as shown in Figure 1A, *inset*. At the pinholes, scattered and out-of-focus fluorescence is blocked like in any confocal microscope. A LP672 long-pass dichroic separates the fluorescence from the IR excitation light. An additional multi-photon emission filter (ET700SP-2P, Chroma) was used to block residual IR excitation light. For detection, the pinhole plane is imaged on to an sCMOS camera chip (PCO Edge 4.2) with a total magnification of ×36. With the current ×5 beam expansion, this optical arrangement results in an image of about 7 mm diameter on the camera chip, corresponding to an effective field-of-view of about 200 μm, which is sampled at 182 nm/px. For image acquisition, the disk is rotated at a constant speed of 5,000 RPM. Due to the four nested spirals, a quarter rotation is sufficient for a homogenous illumination of the sample, so a minimal acquisition time of 3 ms per full frame can be obtained.

The described system (with the exception of the Laser and shutter) is constructed in a monoblock upright microscope system with a voice coil-based objective drive (Figure 1C). A movable mirror is used to switch between 2P-spinning disk fluorescence imaging and a bright-field trans- or reflected light mode (with a *f* = 140 mm TL and a separate USB camera, Point Grey, BFLY-U3-23S6M-C). Having an effective magnification of ×17.5, this alternative optical path can be used to get an overview of the sample and identify regions of interest. The laser, shutter, focus and image acquisition are all controlled by an in-house microcontroller and software (TILL Siam).

### Image Analysis and Quantification

Images were analyzed and displayed using FIJI. For a better visibility of the faint fluorescent signals at greater imaging depths, we used a nonlinear look-up table (*γ* = 0.6) for Figures 4B–E, 5C,D.

**Figure 3:**
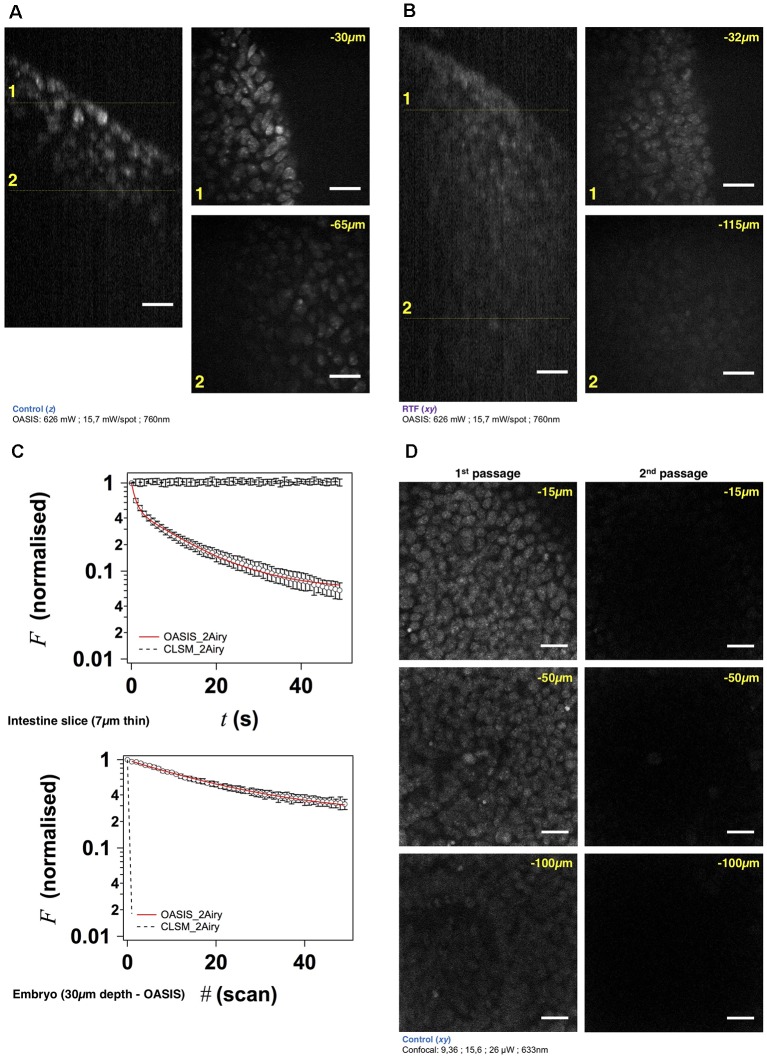
The OASIS microscope outperforms a CLSM point-scanner for 3-D embryo imaging. **(A)** 3-D data set taken with the OASIS in a non-cleared embryo stained with TO-PRO3 (Control). Panels show *xz-* projection of a *z*-stack of images (*right*) and *xy*-planes (*left*) corresponding to the dashed lines at 30 μm (1) and 65 μm imaging depth (2), respectively. Scale-bar, 25 μm. **(B)** same, at 32 μm (1) and 115 μm depth (2), respectively, for an RTF-cleared embryo. Scale-bar, 25 μm, as in **(A)**. **(C)** Representative bleaching curves during continuous acquisitions, from a single *z*-section in—respectively—a thin slice of intestine (*top*) and of TO-PRO3-labeled nuclei (*bottom*) at 30-μm imaging depth in an embryo. **(D)** Representative planes at various imaging depths (15, 50 and 100 μm, respectively) of a 3-D data set (200 planes from the surface to 200 μm, Δ*z* = 1 μm) acquired from a TO-PRO3-labled embryo. The two columns show the sections during the 1st passage (*left*) and during the 2nd passage, after completion of the 1st image stack (*right*). Note the almost complete bleaching prohibiting repetitive volume imaging for the CSLM but not the OASIS microscope. Measured laser powers are given for each depth. Scale-bar, 25 μm.

**Figure 4:**
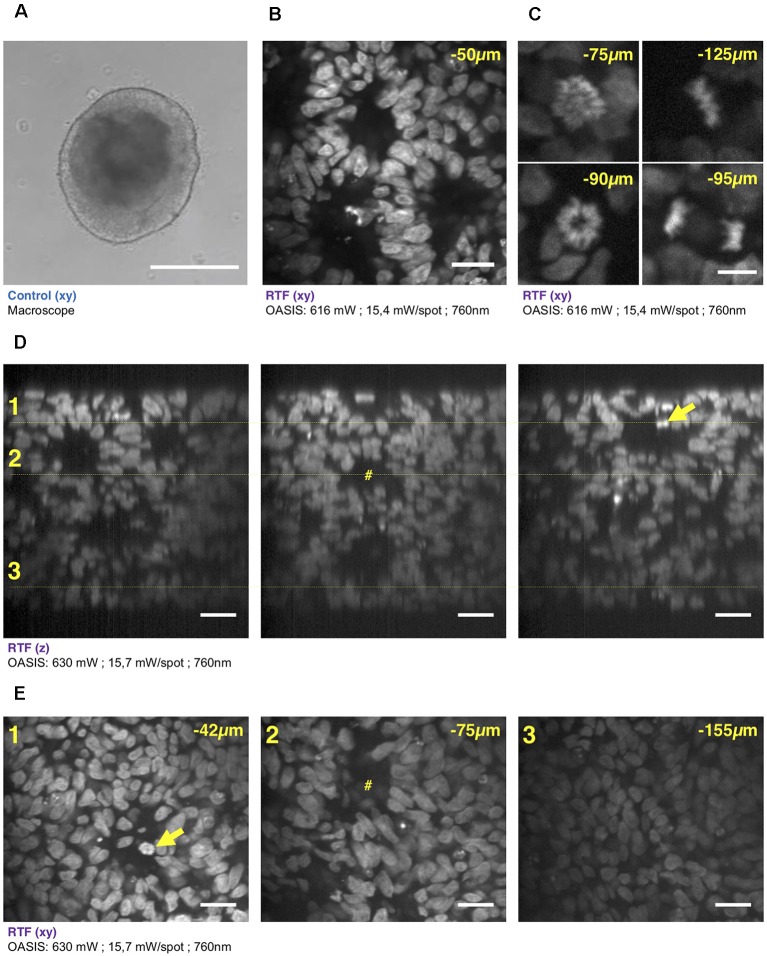
3-D imaging with the OASIS of the fine structure of embryonic bodies (EBs). **(A)** Macrophotography of a day-7 EB; Scale-bar, 300 μm. **(B)** Image acquired in the center of the organoid after TO-PRO3 staining, 50 μm below the surface. Scale-bar, 25 μm. Note the presence of internal round structures with a lumen. **(C)** Zoom on mitotic figures observed in the EB at various depth (*top left*: prophase; *top right*: metaphase; *bottom left*: early anaphase; *bottom right*: late anaphase); stain: TO-PRO3. Scale-bar for all panels, 10 μm. **(D)** *xz*-pro-jections of a *z*-stack acquired across an entire day-7, TO-PRO3-stained EB. Scale-bar, 25 μm. Arrow points to mitosis also visible in panel **(E)**. ^#^Indicates ventricle-like structure also perceived in **(E)**. **(E)** *xy*-sections across the dotted lines shown in **(D)** at imaging depths of, respectively, 42 μm (1), 75 μm (2) and 155 μm (3). Scale-bar, 25 μm. With the exception of **(A)**, all images were acquired after RTF clearing.

**Figure 5:**
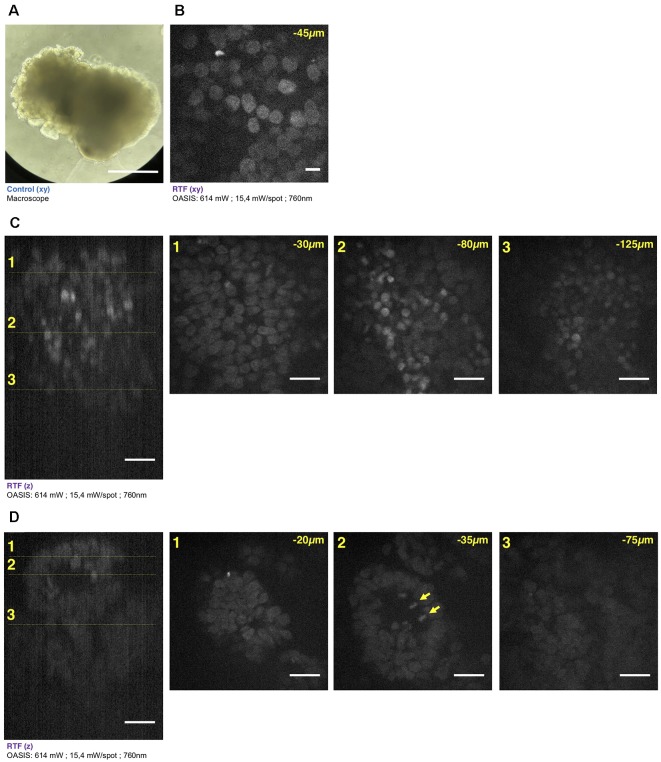
Brain organoid imaging with OASIS. **(A)** Macrophotography of a day 11 brain organoid before clearing and observation on the OASIS microscope; scale-bar: 300 μm. **(B)** Image acquired in the center of the organoid at 45 μm below the surface; stain: TO-PRO3; scale-bar: 10 μm. **(C)** z-reconstruction (left) and xy-acquisition (right) at respectively 30 μm (1), 80 μm (2) and 125 μm (3) below the surface of the organoid; stain: TO-PRO3; scale-bar: 25 μm. Note the change of the morphology of the nuclei in the center of the region of interest when depth increase. **(D)** z-reconstruction (left) and xy-acquisition (right) at respectively 20 μm (1), 35 μm (2) and 75 μm (3) below the surface of the organoid; stain: TO-PRO3; scale-bar: 25 μm. Note the globular structure (neuro-epithelium) that can also be observed on the macroscopy and numerous mitosis inside this structure (arrows). Except in **(A)**, all images were acquired after the clearing of the organoid with the RTF protocol. Note the image quality across the entire imaged volume.

Weber contrast was calculated as *C*_w_ = (*F − B*)/*B*, where *F* and *B* are the fluorescence intensities of the image and background, respectively. *B* was measured as the mean intensity of a cell-free region and *F* corresponded to the mean intensity in a small ROI (18 μm × 18 μm) in the center of the image for Supplementary Figure S4A, and to the whole image in Supplementary Figure S4B. In Supplementary Figure S4A, the sample was inclined respect to the optical axis so that only a small region could be used to quantify the penetration depth.

### Statistics

All results are at least triplicates of three independent experiments and are represented as mean ± SD. Student’s *t*-test was used to compare among experiments. Data were processed and figures prepared using IGOR Pro (Wavemetrics).

## Results and Discussion

### Wide-Field Two-Photon Microscopy at Diffraction-Limited Resolution

With our OASIS system, we retain the in-line geometry of a classical upright microscope with a single objective lens (OL). We introduce a novel spinning-disk concept, rethought and specifically designed for wide-field 2P-excitation microscopy (Figure 1A). Briefly, the expanded and shaped beam of a fs-pulsed infrared laser is focused by an array of micro lenses to produce some 40 evenly lit excitation spots. These spots are imaged by the TL and objective into the sample plane where they are each spaced, on average, by 28 μm (Figure 1A, *inset* ❶). Almost 5,000 micro lenses are arranged in four spirals that scan these spots upon rotation of the disk. At 5,000 rpm (i.e., one turn every 12 ms, much shorter than camera integration times used here) this multi-photon multi-focal microscope generates a virtual light sheet permitting wide-field excitation, direct-view 2P microscopy. Unlike earlier 2P-spinning disk microscopes (Bewersdorf et al., 1998; Egner et al., 2002; Shimozawa et al., 2013) based on the Yokogawa-type dual-disk design, we conceptualized and built a completely different geometry. Where the Yokogawa design requires two disks, one with micro lenses, the other one with confocal pinholes, our design relies on a single disk on which micro lenses and pinholes are arranged on the different faces of the same optical element. In addition, each micro lens comes with its own dichroic mirror; the dielectric coating on the rear face is omitted over a tiny central circular aperture of 60-μm diameter, thus most of the disk is opaque for fluorescence which must arrive focused in the plane of the disk to pass. The use of a single disk is advantageous in view of the large wavelength difference between the near-infrared excitation and the visible fluorescence, which had previously reduced the efficiency of 2P-spinning disk microscopes. However, this simplification comes at a price as it required a complete re-design of the microscope optical path. On the *excitation* side, the micro lenses must be uniformly illuminated with an expanded, collimated, flat-profile fs-pulsed IR laser beam to generate an array of focused spots. On the *collection* side, to pass the pinholes, the detected fluorescence must arrive focused at the level of the pinholes. The required optical path-length difference between excitation and emission light is achieved by introducing a corrective distance element (CDE) in the non-infinity space: while the longer-wavelength excitation light passes straight through the device, the shorter-wavelength fluorescence takes a detour and travels a longer path to produce the desired focal offset, *inset* ❷. This patented scheme critically relies on extremely flat shallow-incidence long-pass dichroic mirrors to preserve the phase front of the beam and maintain the optical resolution, but it has the crucial advantage to be compatible with much bigger objective pupils (and hence larger fields of view and working distances) than the Yokogawa dual-disk design where the dichroic cube is sandwiched between the two disks.

The 2P-excited fluorescence generated in each of the spots is collected through the same objective and imaged onto the tiny pinholes in the dichroic coating of the microlenses (Figure 1A, *inset* ❷). Relay optics then images these pinholes onto a large-format sCMOS camera. With the ×25/1.1NA objective, the resulting pixel size in the sample plane is 182 nm. As the fluorescence is de-scanned at the level of the disk, this detection is partially confocal (the 60-μm pinhole diameter correspond to a confocal aperture of 2 Airy units), so that ballistic and snake-like photons but not scattered fluorescence are collected. This is illustrated by the exponential fluorescence drop with increasing sample turbidity (i.e., equivalent imaging depth). Yet, as a result of the longer wavelength of excitation light compared to 1P-CLSM, the signal drop observed with the OASIS microscope was only half of that observed upon 1-P 633-nm excitation on the ZEISS LSM710, and this although two photons must combine for fluorescence excitation (leading to a squared exponential fall-off; Figure 1B). We can attribute the improved depth penetration of the OASIS uniquely to excitation effects, because stopping down the confocal pinhole of the LSM710 from 2 to 1 Airy units did not measurably alter the fluorescence decay on the CLSM, indicating that at the imaging depths attained, pinhole size effects are minor.

With its small footprint (43 cm by 12 cm) and 22-cm clearance under the objective, the OASIS microscope offers facile access and ample space around the objective, making it an ideal platform for imaging large samples, but also for placing electrodes, application pipettes or external fibers for photostimulation or photochemical uncaging (Figure 1C). As a result of the large chip format of the sCMOS detector, we could implement simultaneous dual-color detection by way of a custom image splitter (Supplementary Figure S1).

With the MaiTai DeepSee, ×4.6 beam expander (BE) and a ×25/NA1.1w low-mag high numerical aperture (NA) dipping objective (Oheim et al., 2001), the OASIS microscope features an effective field of view with a 190 μm image diagonal. An elongated, larger field of 380 μm by 190 μm would be possible, but it would require more micro-lenses to be illuminated with an elliptical beam, which in turn would require a more powerful fs-pulsed laser than ours.

As a wide-field excitation, direct-view imaging system, the OASIS microscope simultaneously offers a fast frame rate, and it resolves tiny subcellular detail over a large field of view. We illustrate the sub-micrometric resolution by imaging the fine tip of a spine from an autofluorescent pollen grain, a popular sample for testing 2P-microscopes (Figure 1D).

For getting a first estimate the optical sectioning capability of our microscope, we recorded the axial (*z-*) intensity profile when focusing at the interface of a green fluorescent Chroma test slide and we quantified Δ*z* by the taking the full-width at half maximum (FWHM) of a Gaussian fitted with the derivative of the *z*-profile (König, 2018; Figure 1E). Repeating the same experiment on the ZEISS LSM710 demonstrates that OASIS offered a 1.3-fold better optical sectioning with the pinhole diameter set to 2 Airy diameter and with a dipping objective having a similar NA (1.1w for the OASIS vs. 1.0w for the ZEISS; Δ*z*_OASIS_ = 2.75 ± 0.02 μm vs. Δ*z*_CLSM, 2Airy_ = 3.47 ± 0.02 μm). In fact, the optical sectioning of the OASIS microscope is close to that of a CLSM with the pinhole stopped down to 1 Airy diameter (Δ*z*_CLSM, 1Airy_ = 2.55 ± 0.02 μm; Supplementary Figure S2). We note that both values are not optimal due to the refractive-index mismatch between the water immersion and the Chroma test slide, but this does not change the factor between the two (neither of these lenses had a correction collar).

The acquisition speed will in practice be limited by the available signal, but the theoretical minimal exposure time is given by the time to obtain a homogeneous illumination of the sample. With a rotation time of 12 ms at 5,000 rpm and four nested spirals, the minimal exposure time is 3 ms corresponding to a time resolution of 6 ms according to Nyquist’s sampling theorem in the temporal domain. Exposure times should be multiples of 3 ms for obtaining homogeneously illuminated field of views. With the 100-fs pulses and <10 mW average laser power, <*P* >, per illumination spot used here, typical exposure times were of the order of 48–120 ms, full-frame, more than one order of magnitude faster than the typical time required for a similarly resolved image with confocal or 2P-scanning microscope.

Taken together, our OASIS 2P-spinning disk microscope offers a larger field, a better depth penetration, a similar if not better spatial resolution and a considerably higher speed than a conventional CSLM.

### RTF-Cleared TO-PRO3-Labeled Embryos As a Test Sample for 1- and 2P Microscopies

Next, we evaluated the performance of the OASIS microscope for 3-D imaging of partially cleared brain tissue. We sought for a stereotypic, sparsely but relatively homogenously labeled thick sample. To allow for a direct comparison between 1P-CLSM and our 2P spinning-disk microscope, this labeling needed to be suitable for both linear- and non-linear excitation. To minimize scattering and improve the depth penetration, we sought a red-excited, deep-red emitting fluorophore. With these constraints in mind we evaluated the nuclear stains TO-PRO3 and Methyl Green (MG), having 1P-fluorescence excitation/emission maxima of 642/661 nm (Bink et al., 2001) and 632/650 nm (Prieto et al., 2014), respectively.

During preliminary experiments in 7-μm thin sections of a fixed (E14.5) mouse embryo, we found TO-PRO3 fluorescence to be 2.1-fold brighter than that of MG upon 633-nm excitation. We observed an even larger intensity ratio (×5.5) upon 2P-excitation at 760 nm (Figure 2A). Although non-linear excitation of TO-PRO3 at 1,100 nm has been reported (Smith et al., 2012; Ricard et al., 2018) our own measurement of the 2P-action spectra revealed peak excitations at 760 and 750 nm for TO-PRO3 and MG, respectively (Figure 2B). These shorter wavelengths are within the tuning range of the Ti:Sapphire laser and they minimize thermal damage from near-infrared absorption and focal heating (Schmidt and Oheim, 2018), a particular concern for the multi-spot excitation scheme used here.

We next optimized the tissue clearing procedure. Among the available methods (for review, see Richardson and Lichtman, 2015), we focused on TDE (Aoyagi et al., 2015), Clear^T2^ (Kuwajima et al., 2013) and RTF clearing (Yu et al., 2018). The rationale was that these methods require only short clearing episodes and they use solvents compatible with dipping objectives. Mouse embryos were most transparent with TDE (60%), followed by RTF and, by far, Clear^T2^, for which the tissue was even more opaque than the non-cleared control (probably due to volume shrinkage; Figure 2C).

In clearing protocols, transparency is one issue, fluorescence preservation another. Depending on the very method used, the observed fluorescence loss was dramatic, with a 99.9% and 92% attenuation of TO-PRO3 fluorescence following Clear^T2^ and RTF clearing, respectively (Figure 2D). Increasing the laser power by a factor of 20 allowed us to acquire confocal images of TO-PRO3 stained nuclei in slices of RTF-cleared embryos (Figure 2E), whereas TDE clearing attenuated the fluorescence to undetectable levels (Supplementary Figure S3). To develop an order-of-magnitude idea of the laser powers required for obtaining similar signal-to-noise levels with the OASIS and CSLM, we finally compared images acquired upon 1- (at 633 nm) and 2-excitation (at 760 nm) of TO-PRO3 labeled nuclei in a thin section of RTF cleared mouse embryo. At equivalent confocal aperture of 2 Airy, we found a factor of ×4,000 between linear and non-linear excitation (2 μW vs. 8 mW/spot, respectively).

Based on these results, we opted for TO-PRO3 nuclear staining and RTF clearing for directly comparing the performance of confocal and OASIS microscopes.

Many questions in neurobiology require at high-resolution imaging of large sample volumes. The growing field of 3-D cultures, EBs and minibrains is no exception, as many basic neuroanatomical questions remain open. For many developmental biology and neurobiology laboratories, that are not necessarily experts in 2P- or light-sheet microscopies, the single-spot scanning confocal laser scanning microscopy (CLSM) remains the major workhorse. Due to its simplicity and ease of use, our OASIS microscope aims precisely at such end-users, and we, therefore, compared the OASIS with a commercial ZEISS LSM710 confocal, an instrument present on many imaging platforms.

### Faster and Less Invasive Acquisition of 3-D Image Stacks Than a CLSM

At 250-nm lateral and micrometric axial sampling and with typical pixel dwell times of 1 (10 μs), sequential single-spot scanning requires 4 (40) ms, 4 (40) s and more than 1 (10) for the acquisition of 3-D image stacks from cubes of 10 μm, 100 μm and 1 mm side-length, respectively. Parallelizing both the excitation and emission detection, as with our OASIS microscope is expected to considerably speed up the imaging of such large data sets.

Using TO-PRO3 nuclear staining as a proxy, we acquired *z*-stacks of images in RTF-cleared mouse embryos. Achievable imaging depth in TF-cleared samples (Figure 3B and Supplementary Figure S4A) were three-fold increased compared to non cleared mouse embryos (Figure 3A and Supplementary Figure S4A), 90 μm vs. 30 μm. Next, we evaluated, at similar signal-to-noise ratios of the resulting images, the photobleaching with the OASIS (at 760-nm 2P-excitation) vs. the ZEISS LSM710 spot scanning confocal (at 633-nm 1P-excitation). We first compared 2P-large-field and confocal-scanning excitations at shallow imaging depths, by continuously recording images of the same region of interest (ROI) for a 7-μm intestine slice. At the same initial signal-to-noise ratio for either microscope, we observed a ~3%-intensity loss after the first full-frame with the OASIS microscope (*t*_exp_ = 480 ms per image, 15.7 mW/spot for the OASIS), whereas the signal remained relatively stable after a single confocal scan. Fitting a monoexponential with the OASIS bleaching data revealed a 1/e (37%) loss of fluorescence every 21 frames (Figure 3C). Thus, as expected, the high peak-powers required for non-linear excitation result in a significantly higher bleaching when imaging single planes in superficial tissue layers. However, for 3-D volume imaging of thicker sections, confocal microscopy rapidly produced much faster photobleaching than the OASIS because the entire volume is exposed throughout the acquisition. Also, the CLSM required higher and higher laser powers to maintain image contrast at greater imaging depths; in fact, acquiring the first complete *z*-stack with the CSLM attenuated the TO-PRO-3 fluorescence so much that a second volume acquisition was impossible (Figure 3D).

We attribute the much higher volume photobleaching upon 1P confocal imaging in non-cleared embryos to four reasons: (i) tissue scattering at 633 nm was roughly double that of near-infrared light. The exponential scattering losses of excitation photons must be compensated for by exponentially increasing the excitation powers with increasing imaging depth; (ii) as a consequence of linear (1P) excitation, off-focus excitation of fluorophores located above and below the imaged plane causes bleaching, too, i.e., at any plane, bleaching occurs throughout the entire tissue volume while only one plane is imaged; (iii) although not contributing to imaging, the scattered 1P excitation light nevertheless excites (out-of-focus) fluorescence, which—in addition to the ballistic out-of-focus excitation—additionally results in volume photobleaching. Non-linear (2P) excitation, on the other hand, confines both fluorescence excitation and photobleaching to the focal plane, with the result of better preserving the sample outside the plane which is actually imaged; and (iv) image acquisition was >4-times faster on the OASIS compared to confocal scans (480 ms/image vs. 1.815 s/image for a similar image contrast), reducing the overall exposure of the sample.

We note that the better performance of the OASIS concept comes essentially from the effect 2P-excitation confinement, because on the fluorescence collection side, at 2 Airy, both instruments should perform similarly. Finally, not only taking into account the loss of signal but also Weber contrast, we found a three-fold larger effective depth penetration for the OASIS microscope compared to the CLSM (Supplementary Figure S4A).

Taken together, the OASIS microscope combines the advantages of 2P-excitation and excitation and emission parallelization and therefore excels over the ZEISS LSM710. It achieves higher *z*-resolution, affords less photobleaching in 3-D samples and considerably speeds up data acquisition, thus allowing a more efficient and less invasive volume imaging. These properties make it an ideal instrument for imaging EBs, organoids, 3-D cultures and small model organisms.

### Fast Volume Acquisition From Mouse Embryonic Bodies and Brain Organoids

We continued our comparison of the OASIS against the CLSM by imaging day-7 EBs (Figure 4A), again stained with TO-PRO3. Images acquired at different depths displayed rounded structures with *luminae* inside tissue and revealed strong mitotic activity (Figure 4B). The rounded structures presumably correspond to neuroepithelial-like structures that are readily formed within EBs, indicating the inherent ability of the ectoderm to differentiate into neural lineages (Ying and Smith, 2003). The sub-μm resolution of the OASIS microscope allowed us the detailed characterization of the different mitotic figures throughout the entire 160-μm thickness of the EB, which was impossible on the CLSM (Figure 4C).

Acquiring 3-D image stacks at a *z*-spacing of 0.5 μm (Supplementary Figure S4B) allowed us reconstructing the complete EB volume and realizing high-resolution projections along the orthogonal axes (Figure 4D), revealing fine structural detail, including mitosis across its entire volume (Figure 4E). Volume imaging of entire EBs was almost 4-times faster with the OASIS compared to the CLSM (a 200-μm *z-*stack with a 0.5-μm z-spacing required 3′12″ vs. 12′6″), with no detectable photobleaching on the OASIS. Together, these features make the OASIS microscope an ideal setup for the detailed 3-D characterisation of EB development.

Similar if not larger *z*-stacks were acquired a from RTF-cleared day-11 brain organoids (Figure 5A). At this early developmental stage, the neuroepithelium has been induced and forms buds that undergo 3-D growth within the Matrigel droplets (Lancaster and Knoblich, 2014). Our observations highlight strong morphological modifications during this tissue expansion. A recurrent feature was that TO-PRO-3 labeled nuclei were rounded just below the surface of the brain organoids, whereas polymorph and diamond-shapes prevailed at greater imaging depths (Figure 5B). Also, the cell density and nuclear labeling changed markedly with depth. Orthogonal planes revealed compact groups of nuclei with stronger fluorescence (Figure 5C), as well as cavities and rounded structures with a neuroepithelium-like shape. As before in the EBs, the resolution of the OASIS microscope allowed us to detect the presence of mitotic figures at the luminal side (Figure 5D). Typical achievable imaging depths were now around 200 μm, reflecting the densification and opacification of the tissue during the development of an EB towards a brain organoid.

With its large field-of-view, increased depth penetration, low photobleaching and greater speed compared to the CSLM, the OASIS optical scheme lends itself ideally to the observation of entire EBs and brain organoids. Thin optical sections can be studied at depth with sub-μm lateral resolution, without mechanical slicing. 3-D imaging at subcellular resolution can also be achieved with expansion microscopy, in which a polymer network is introduced into cellular or tissue samples, and this network physically expanded using chemical reactions to increase the size of the imaged brain structures relative to the available microscope resolution (Wassie et al., 2018; Gao et al., 2019). Complementary to and compatible with this type of microscopy, the OASIS microscope is an interesting platform for such expansion strategies. The reduced complexity compared to a classical light-sheet microscope, its compact mono-block design and comparable ease-of-use make it an ideal companion for functional neuroanatomy. The ongoing integration of a compact, fixed-wavelength high-power fs-pulsed laser into this package will make the OASIS a unique, portable, alignment-free bench-top 2P-microcope.

## Conclusion

Work on brain organoids offers several distinctive advantages over classical disease models: (i) derived from patient fibroblasts, they raise fewer concerns than animal experimentation and work on human explants (see, however, Farahany et al., 2018, for an emerging awareness of the ethical issues associated with these 3-D cultures); (ii) they avoid the limitations of animal models that are often only a poor proxy of human pathology; (iii) they allow studies of rare or sporadic cases, for which genetic models are missing; and (iv) they allow observing the onset of the disease during the early steps of the brain development, opening opportunities for studies that would be impractical or unacceptable on human embryos or infants.

For this rapidly growing field of applications, the OASIS 2P microscope is a compact, versatile and cost-efficient 2P wide-field research instrument allowing imaging of hiPSC cultures, EBs and brain organoids.

## Ethics Statement

All experimental procedures were performed in accordance with the French legislation and in compliance with the European Community Council Directive of November 24, 1986 (86/609/EEC) for the care and use of laboratory animals. The used protocols were approved by the local ethics committee.

## Author Contributions

AD, CS and RU designed and conceptualized the OASIS prototype. BD and PD generated iPSCs, EBs and brain organoids. AD, BD, IR, CR, MB and MO performed the experiments. MO wrote the article with contributions from all authors.

## Conflict of Interest Statement

RU is the founder and CEO of TILL.id, AD and CS are employees of TILL.id.

## Footnotes

^1^The “*brain in a vat*” or “*brain in a jar*” is a scenario used in a variety of *Gedanken experiments* intended to draw out certain features of human conceptions of knowledge, reality, truth, mind, consciousness and meaning. It is an updated version of René Descartes’ evil demon thought experiment. It has been extensively used by Hilary Putnam (*Reason, Truth and History*, 1981), in an argument inspired from Roald Dahl’s short story, «*William and Mary*» (1959).

^2^https://idisco.info/idisco-protocol/
