# Supplementary material for: Fast 3-D Imaging of Brain Organoids With a New Single-Objective Planar-Illumination Two-Photon Microscope
Source: Front Neuroanat. 2019 Aug 20;13:77. doi: 10.3389/fnana.2019.00077 (PMC6710410; doi:10.3389/fnana.2019.00077)
Supplement: Supplementary file 1 [file Data_Sheet_1.PDF]

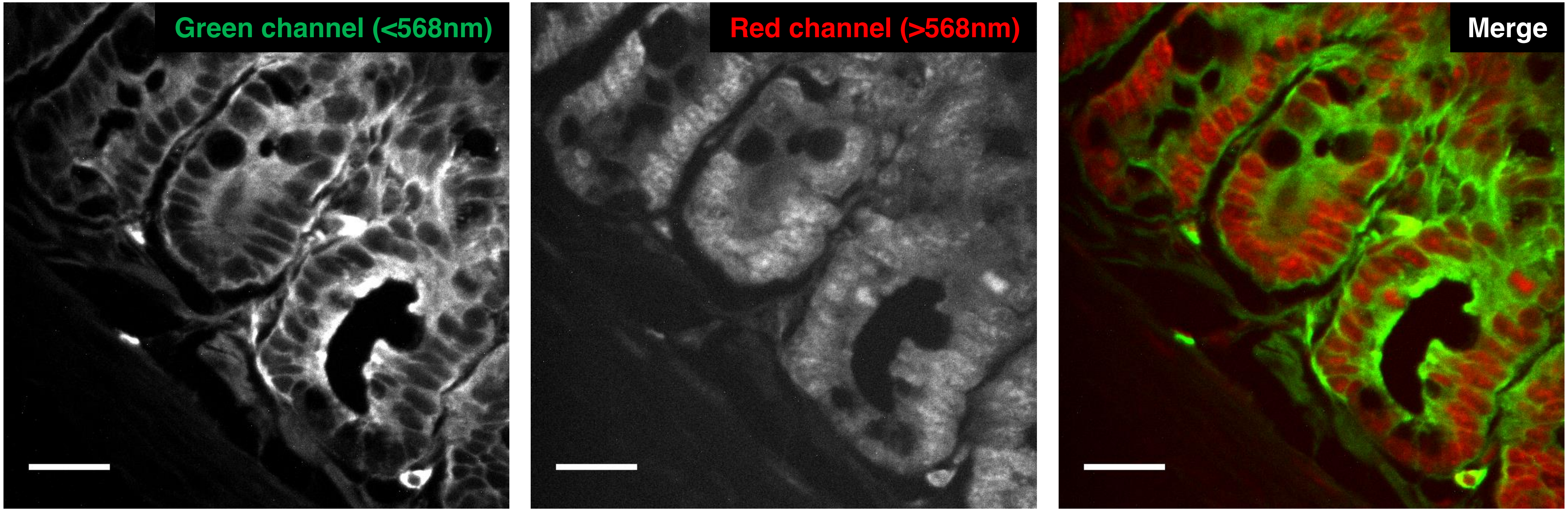

Control (*z*)

OASIS: 620 mW ; 15,5 mW/spot ; 760nm

Figure S1

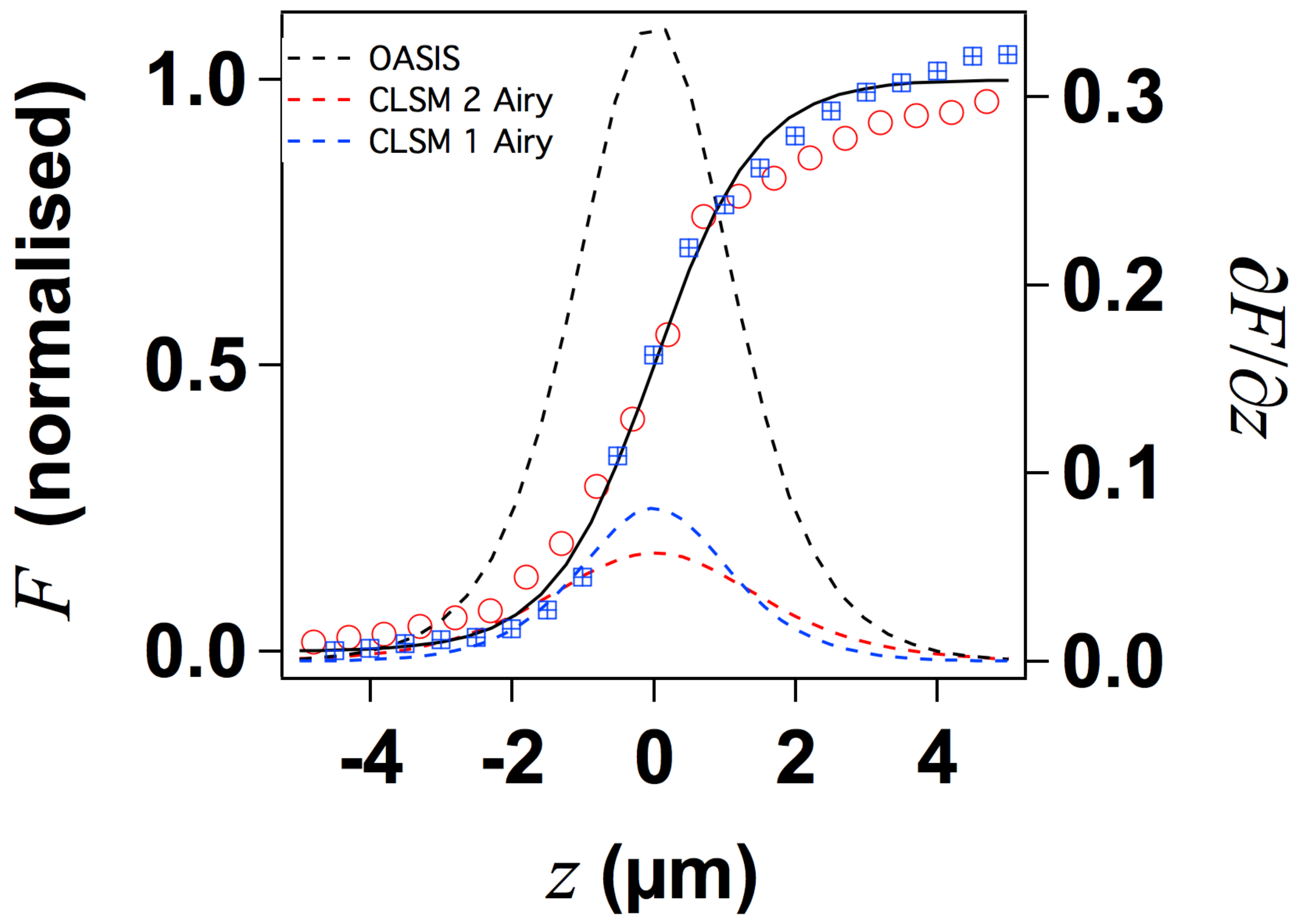
OASIS
CLSM 2 Airy
CLSM 1 Airy
F (normalised)
1.0
0.5
0.0
∂F/∂z
0.3
0.2
0.1
0.0
-4
-2
0
2
4
z (μm)

Figure S2

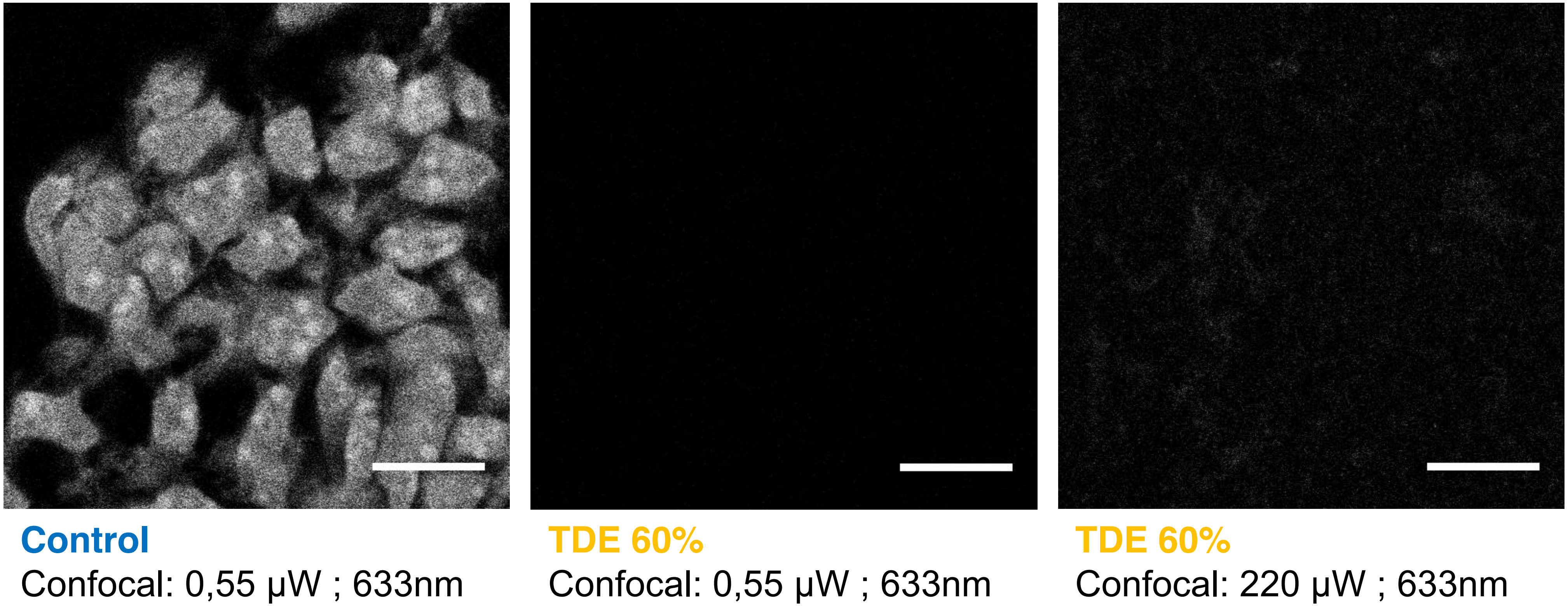
Control
Confocal: 0,55 µW ; 633nm
TDE 60%
Confocal: 0,55 µW ; 633nm
TDE 60%
Confocal: 220 µW ; 633nm

Figure S3

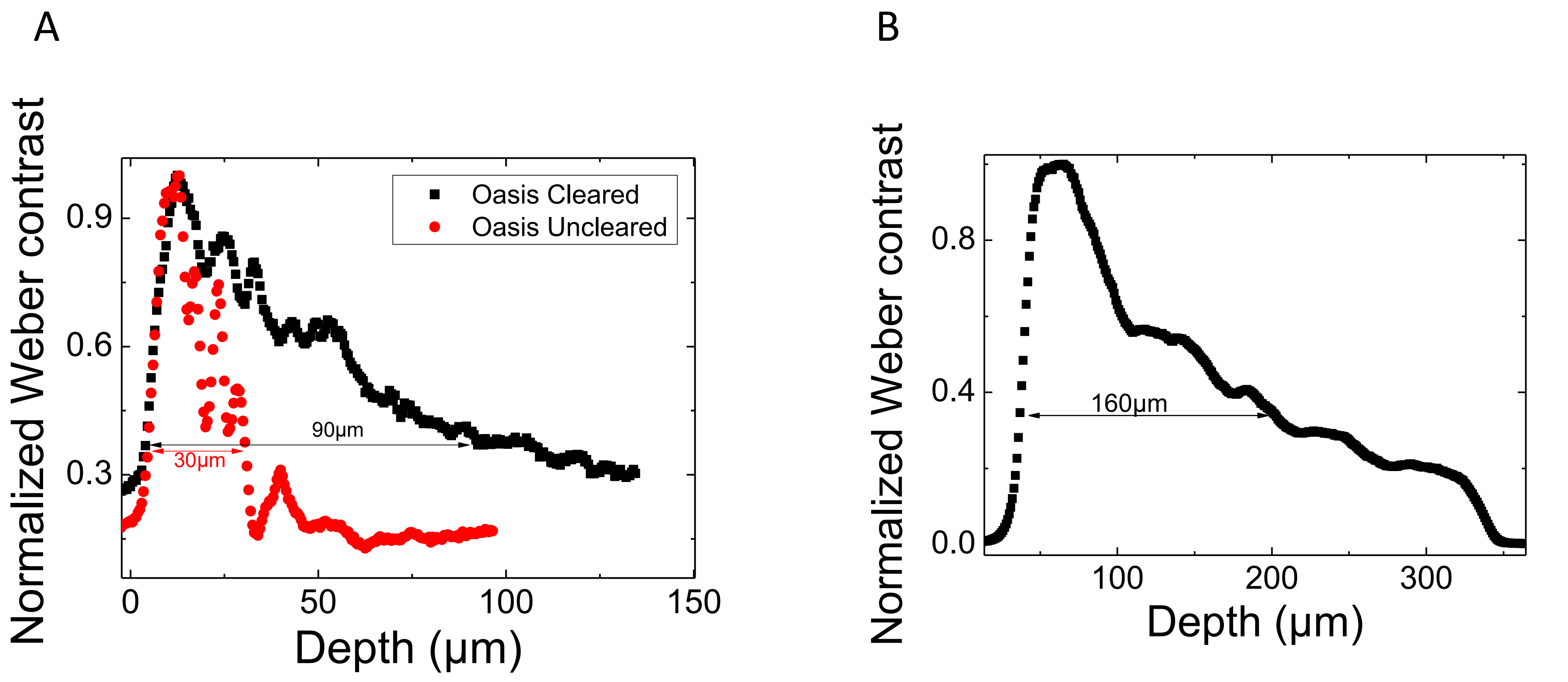
A
B
Normalized Weber contrast
0.3
0.6
0.9
0
50
100
150
Depth (μm)
Oasis Cleared
Oasis Uncleared
90μm
30μm
0.0
0.4
0.8
100
200
300
160μm

Figure S4
