# Supplementary material for: Fast 3-D Imaging of Brain Organoids With a New Single-Objective Planar-Illumination Two-Photon Microscope
Source: Front Neuroanat. 2019 Aug 20;13:77. doi: 10.3389/fnana.2019.00077 (PMC6710410; doi:10.3389/fnana.2019.00077)
Supplement: Supplementary file 2 [file Data_Sheet_2.docx]

**SUPPLEMENTARY MATERIAL**

**Fast, multicolor 3-D imaging of brain organoids with a new single-objective two-photon virtual light-sheet microscope**^£,%^

Irina Rakotoson,^1,2,3,4+^ Brigitte Delhomme,^1,2,3+^ Philippe Djian,^1,2,3+^ Andreas Deeg,^5^ Maia Brunstein,^1,2,3^ Christian Seebacher,^5^ Rainer Uhl,^5^ Clément Ricard,^1,2,3,§^ and Martin Oheim^1,2,3,§,🖂^

^1^CNRS UMR 8118, Brain Physiology Laboratory, Paris F-75006, France

^2^Fédération de Recherche en Neurosciences CNRS FR 3636, Paris F-75006, France

^3^Faculté de Sciences Fondamentales et Biomédicales, Université Paris Descartes, PRES Sorbonne Paris Cité, F-75006 Paris, France

^4^Master programme: BCPP (Biologie cellulaire physiologie et pathologie), spécialité Neurosciences

^5^TILL.id, Am Klopferspitz 19, D-82153 Planegg/Martinsried, Munich, Germany

^*^*corresponding author*

Martin Oheim

[martin.oheim@parisdescartes.fr](mailto:martin.oheim@parisdescartes.fr)

^+^co-first authors

^§^co-last authors

^%^Elements of this work has been published in the Master thesis of I.R. [[1](#_ENREF_1)]

^£^This paper is part of a Frontiers Research Topic "*Light Sheet Microscopy: from Technological Developments to Prospective Applications*" (Guest Editors: Stéphane Pagès, Laura Batti, Corinne Brana, Anthony Holtmaat).

The authors declare not conflict of interest. Rainer Uhl is the founder and owner of TILL.id, Andreas Deeg and Christian Seebacher are employees of TILL.id.

The funders of this study had no hands on the outcome or interpretation of the obtained results.

**SUPPLEMENTARY EXPERIMENTAL PROCEDURES**

***Milk turbidity assay****.* A green fluorescent test slide (Chroma) was imaged on the OASIS microscope upon 920-nm excitation with a laser power of 427 mW (10.7 mW/spot) at the objective. Fluorescence was collected < 568 nm, either using water as an immersion medium or else water containing different percentages of semi-skimmed dairy milk, (Monoprix, 0, 1, 2, 3, 4 and 5%). For each concentration, the mean fluorescence was measured in a ROI located at the center of the image and the resulting fluorescence *F*, for each concentration, averaged over four acquisitions, and log(*F*) plotted *vs.* concentration. Likewise, for CLSM, we used 488-nm acquisition and 0.52 µW (1.04 µW) at 2 (1) Airy Unit. On the 1P-confocal, fluorescence was collected in the band between 500 and 565 nm.

***Immunohistochemistry***. 2 cm of adult mouse *jejunum* (small intestine) were fixed overnight in 4% paraformaldehyde (Merck) at 4°C and pH 6.9, cryo-protected by successive incubations in, respectively, 15% and 30% saccharose until the sample sank to the bottom of the tube, then embedded in OCT and transversally cryo-sectionned to 7-µm slices. Slices were conserved at -80°C before being processed for indirect immunofluorescence. For staining, slices were rehydrated, permeabilized with 0,2% Triton in PBS during 15 min, and blocked with 5% bovine serum albumin (BSA) in PBS. We used a mouse monoclonal primary antibody against E-cadherin (1/500 Santa Cruz sc-21791) and secondary goat anti-mouse IgGs, conjugated to AlexaFluor488 (green channel). In addition, we counterstained the slices with TO-PRO3 (1/1000) for nuclear labeling (red channel). Slices were then mounted with aqueous base medium, (Immu-Mount, pH 8.0-8,8, refractive index *RI* = 1.586, Shandon).

***xyz-resolution measurements***. Lateral (*xy*-) resolution measurements were realized on auto-fluorescent thorny pollen grains. The mean power at the objective was 43 mW (1 mW/spot) at 760 nm. We drew fluorescence intensity profiles across a spine to measure their apparent size. Axial (*z*-) sectioning was estimated using the technique of König [[32](#_ENREF_32)] by acquiring a *z-*stack (Δ*z* = 0.5 µm) from a green-fluorescent test slide (Chroma), measuring the axial-intensity profile, and fitting a Gaussian function with the derivative of the axial fluorescence profile. The measured full-width at half-maximum (FWHM, equivalent to the 10-90% intensity range) was taken as a proxy for the *z*-resolution.

***Two-photon action spectra****.* Action spectra of red-emitting nuclear dyes were measured from 7-µm embryonic slices, stained either with TO-PRO3 or Methyl Green. Images were acquired across the tuning range of the Ti:Sapph laser (750 nm, and in steps of 20 nm in between 760 nm and 1040 nm) on the OASIS microscope using constant power. 2PEF was collected above 568 nm. 10 regions of interest (ROI) centered on stained nuclei were selected, and their mean fluorescence intensity was measured. To correct for background, 10 ROIs in unstained areas were selected and the average background subtracted from the nuclear fluorescence. Finally, for each excitation wavelength, the fluorescence intensity was normalized to the average laser power <*P*>.

***Fluorescence-intensity measurements****.* To compare fluorescence intensity among nuclei under different staining and clearing conditions (TO-PRO3 vs. Methyl Green; TO-PRO3 staining in control vs. TDE-, ClearT2- and RTF-cleared samples), the mean fluorescence intensity was extracted from 10 different nuclei. For each ROI, the mean background intensity (measured in 5 distinct ROI) was subtracted. Data displayed in the bar-plots shows the means of these 10 ROI ± SD.

***Photobleaching measurements on embryos.*** *OASIS.* A times-lapse series of 50 images (*t*_exp_ = 480 ms) was acquired at 30-µm imaging depth in a non-cleared, TO-PRO3-stained embryo. <*P*> at the objective was 626 mW (i.e., 15.7mW/spot), *λ*_ex_ = 760 nm. We selected four TO-PRO3-stained nuclei and plotted the evolution with time of their mean fluorescence, normalized to (*F*/*F*_0_)(*t*) = *F*(*t*)/*F*(*t* = 0), after background subtraction.

***Photobleaching experiments on z-stacks****. Confocal laser scanning microscopy* (*CSLM* ). 2 *z*-stacks (0 to 200 µm, 0 denoting the surface; *Δz* = 1 µm) were successively taken in a non-cleared embryo stained with TO-PRO3. *λ*_ex_ = 633 nm. To compensate for excitation-scattering losses, the HeNe laser power was increased with *z* so as to maintain the same signal-to-background ratio (Powers are indicated in the legend of the figure). Data is represented as (*F*/*F*_0_)(*t*), after background subtraction.

***Photobleaching experiments on a 7µm-thick slice of intestine.*** *OASIS.* Fifty images (*t*_exp_ = 480 ms) were continuously taken in an 7µm-thin intestine slice stained with TO-PRO3. Mean power at the objective was 638 mW (16 mW/spot), *λ*_ex_ = 760 nm. Four stained nuclei were selected as ROIs and their mean fluorescence was extracted for each time point. Data is represented as (*F*/*F*_0_)(*t*), after background subtraction. *CLSM.* Fifty images (pixel-dwell time of 0.58 µs, i.e., 546 ms/image) were continuously acquired from a 7-µm intestine slice, stained with TO-PRO3. Mean power at the objective was 0.5 µW, *λ*_ex_ = 633 nm. Fluorescence was collected in the 646-725 nm band, and the pinhole diameter set to 2 Airy, to match the OASIS setup. Four stained nuclei were selected as ROIs and their mean fluorescence was extracted for each time point. Data is represented as (*F*/*F*_0_)(*t*), after background subtraction.

**List of abbreviations**

2P - two-photon

3-D - three-dimensional

AOI - angle of incidence

BSA - bovine serum albumin

CLSM - confocal laser scanning microscope

DMEM - Dulbecco’s modified Eagle medium

EB - embryonic body

FWHM - full-width at half maximum

hiPSC - human inducible pluripotent stem cell

LP - long-pass (filter)

MG - Magnesium Green

NA - numerical aperture

OASIS - On-axis 2-photon light-sheet generation *in-vivo* imaging system

OCT - opimal cutting temperature

PBS - phosphate-buffered solution

PEG - polyethylene glycol

RI - refractive index

ROI - region of interest

RTF - Rapid clearing method based on Triethanolamine and Formamide

sCMOS - scientific Complementary Metal Oxide Semiconductor

SD - standard deviation

SPIM - selective-plane illumination

TDE - 2,2’-thiodiethanol

TO-PRO-3 - a carbocyanine monomer nucleic acid stain with red excitation and far-red fluorescence (642 nm/661 nm) similar to Alexa Fluor 647 or Cy5. It is among the highest-sensitivity probes for nucleic acid detection.

**LEGENDS TO SUPPLEMENTARY FIGURES**

**Figure S1** *Two-photon wide-field dual-color imaging with the OASIS.* Mouse small intestine slice stained with TO-PRO3 and immunostained against E-Cadherin, observed with the OASIS microscope. *Left*: E-Cadherin immunolabeling in the green channel (<568nm); *center*: TO-PRO3 staining in the red channel (>568nm); *right*: color merge. Scale-bar, 20 µm.

**Figure S2** *z-sectioning OASIS vs. confocal.*  Axial-intensity profiles (*solid* line) measured from a *z*-stack of images acquired from a green fluorescent Chroma slide, and its derivative *dF*/*dz* (*dashed*). The FWHM of the derivative, equivalent to the 10-90% intensity range, was taken as axial sectioning capacity *Δz* of the OASIS (*black*), and of the ZEISS LSM710 confocal microscope, the latter with the confocal pinhole set to a diameter of 2 (*red*) and 1 Airy. In addition to having a better *z*-sectioning, the OASIS also features a steeper slope of the derivative (amplitude of the dashed curves), which translates into a better contrast around the focal region.

**Figure S3** *TDE clearing reduces TO-PRO3 staining to undetectable levels.* 1P confocal sections from an E14.5 embryo labeled with TO-PRO3 without clearing (Control, *left*) and after clearing with TDE60% (RTF, *middle* and *right* panels). For the middle panel, we used the same laser power as in control, for the right panel full power, indicating the impossibility to recover fluorescence even at the highest available laser power. Scale-bar: 10µm.

**Figure S4** *Normalized Weber contrast as a function of depth.* **A.** Normalized Weber contrast along *z* measured from a stack of images acquired on the OASIS in RTF-cleared (*black*) and uncleared embryos (*red*). **B.** Same, for an RTF-cleared embryonic body. Arrows indicate 1/*e* intensity loss.
